# Genotoxicity of titanium dioxide nanoparticles and triggering of defense mechanisms in *Allium cepa*

**DOI:** 10.1590/1678-4685-GMB-2018-0205

**Published:** 2019-06-27

**Authors:** Ronaldo dos Santos, Taynah Vicari, Samuel A. Santos, Karoline Felisbino, Ney Mattoso, Bruno Francisco Sant’Anna-Santos, Marta Margarete Cestari, Daniela Morais Leme

**Affiliations:** 1 Department of Genetics, Universidade Federal do Paraná (UFPR), Curitiba, PR, Brazil; 2 Department of Plant Pathology, Universidade Federal de Viçosa (UFV), Viçosa, MG, Brazil; 3 Department of Physics, Universidade Federal do Paraná (UFPR), Curitiba, PR, Brazil; 4 Department of Botany, Universidade Federal do Paraná (UFPR), Curitiba, PR, Brazil

**Keywords:** Chromosomal aberrations, micronuclei, nucleolar alterations, cellular alterations, nanoparticle phase-change

## Abstract

Titanium dioxide nanoparticles (TiO_2_NPs) are widely used and may impact the environment. Thus, this study used a high concentration of TiO_2_NP (1000 mg/L) to verify the defense mechanisms triggered by a plant system – an indicator of toxicity. Furthermore, this study aimed at completely characterizing TiO_2_NP suspensions to elucidate their toxic behavior. TiO_2_NPs were taken up by meristematic cells of *Allium cepa*, leading to slight inhibition of seed germination and root growth. However, severe cellular and DNA damages were observed in a concentration-dependent manner (10, 100, and 1000 mg/L). For this reason, we used the highest tested concentration (1000 mg/L) to verify if the plant cells developed defense mechanisms against the TiO_2_NPs and evaluated other evidences of TiO_2_NP genotoxicity. Nucleolar alterations and plant defense responses (*i.e.*, increased lytic vacuoles, oil bodies and NP phase change) were observed in meristematic cells exposed to TiO_2_NP at 1000 mg/L. In summary, TiO_2_NPs can damage the genetic material of plants; however, plants displayed defense mechanisms against the deleterious effects of these NPs. In addition, *A. cepa* was found to be a suitable test system to evaluate the cyto- and genotoxicity of NPs.

## Introduction

Titanium dioxide nanoparticles (TiO_2_NPs) are among the most used manufactured nanomaterials, being produced in thousands of tons per year around the world (Robichaud *et al.*, 2009). This NP occurs in three forms in nature: anatase, brookite, and rutile (Clement *et al.*, 2013). The frequent use of products containing TiO_2_NPs has been associated with inappropriate disposal of domestic and industrial effluents, which may result in the release of this NP into the environment. Thus, there is an emerging concern about the environmental impacts caused by NPs (Monica and Cremonini, 2009), in particular TiO_2_NPs.

Considering that higher plants are suitable models for assessing environmental toxicants and plants are fundamental organisms of all ecosystems, phytotoxicity studies become relevant because: (1) plants are considered the basis of the food chain; (2) plants provide the oxygen necessary for life; and (3) plants are distributed in different environments (terrestrial and aquatic). Thus, damages to plants can generate an imbalance in the ecosystems, emphasizing the importance of phytotoxicity studies (Ma *et al.*, 2010). Within this context, there is a concern about the toxicological impacts of NPs on living organisms, such as plants – target organisms of NPs through different environment compartments (air, water, and soil).

The toxic effects of TiO_2_NPs on plants have been reported in the literature (Ghosh *et al.*, 2010; Klancnik *et al.*, 2010; Pakrashi *et al.*, 2014). However, these studies considered only the nanopowder form, without an adequate characterization of TiO_2_NPs suspensions, fundamental to understanding the internalization, bio-uptake, and the behavior of the TiO_2_NPs inside the cells.

*Allium cepa* is an efficient plant system that has some advantages over other higher plants, in particular regarding its convenient chromosomal features that allow the evaluation of genetic damages. Genotoxic effects of different contaminants have already been reported in the literature using *A. cepa* (Grisolia *et al.*, 2005; Rodrigues *et al.*, 2010; Dourado *et al.*, 2017), including the identification of the mechanisms of action (Fiskesjo, 1985; Leme and Marin-Morales, 2009; Klancnik *et al.*, 2010; Pakrashi *et al.*, 2014).

Moreover, plant systems allow the performance of complementary analysis. Ag-NOR banding technique is a new method to detect a genotoxicity biomarker used in *A. cepa* that has shown to be an efficient tool for studying these effects caused by contaminants (Mazzeo and Marin-Morales, 2015). These authors reported changes in Ag-NOR banding that may suggest aneugenic effects or increased genomic activity. Furthermore, Zhao *et al.* (2016) observed a cellular defense mechanism and morphological damages (*e.g.,* ruptures of the plasma membrane, the appearance of oil bodies, and changes in the vacuoles).

Thereby, this study aimed to evaluate the toxic potential of TiO_2_NPs in higher plants to provide knowledge about the cellular responses after TiO_2_NPs exposure. For this purpose, several biomarkers of toxicity in *A. cepa* test system (*e.g.*, germination rate and root development, morphological and DNA damages, nucleolar alterations) were used. In addition, selected area electron diffraction (SAED) analysis was carried out to verify NP phase-change as a plant defense mechanism.

## Material and Methods

### Chemical characterization of the titanium dioxide nanoparticle suspensions

The TiO_2_NP (powder) was purchased from Sigma-Aldrich^®^ (CAS nº 1317-70-0), with physical characteristics being a particle size 21 nm (TEM), ≥99.5% trace metal basis, and 100% anatase. Morphological characteristics of the TiO_2_NP powder were also determined to verify the crystalline structure by X-ray diffraction, the specific surface area by Brunauer-Emmett-Teller Theory (BET), and the surface chemistry using the X-ray photoelectron spectroscopy (XPS).

The TiO_2_NP was evaluated regarding its toxic potential using the *Allium* test system at 10, 100, and 1000 mg/L. The choice of the highest concentration used in this study was defined based on other studies (Klancnik *et al.*, 2010; Zhu *et al.*, 2010). TiO_2_NP suspensions were prepared in ultrapure water and dispersed in an ultrasonic water bath (42.000 Hz, 160 W) for 30 min immediately before the start of the bioassays with *A. cepa*. The highest concentration (1000 mg/L) was also chosen to observe if the cells developed defense mechanisms, what would be an indication of toxicity, and to observe the internalization, bio-uptake, and behavior of the TiO_2_NPs inside the cells.

The TiO_2_NPs suspensions were also analyzed. The characterization of these suspensions was performed by the Zetasizer^®^ Nano Series ZS90 (Malvern Instruments, Worcestershire, UK) to determine the average particle size (dynamic light scattering - DLS), polydispersity index and zeta potential (Laser Doppler Velocimetry and electrophoresis - LDV). We considered an angle of 90º and a wavelength of 633 nm (Malvern, 2015).

A complementary analysis of the NPs structure was performed by the selected area electron diffraction (SAED) technique, as described below).

### Test system and exposure condition

One hundred *A. cepa* seeds (2n = 16 chromosomes) of the same batch and variety (the “baia periform” onion) were submitted to germination with the TiO_2_NP suspensions (10, 100, and 1000 mg/L), ultrapure water as negative control (NC), 10 mg/L of methyl methane sulfonate (MMS; Sigma-Aldrich, CAS 66-27-3) as positive control (PC) for cyto- and genotoxicity testing, and zinc sulfate heptahydrate (Sigma-Aldrich^®^, CAS 7446-20-0) at 6 mg/mL (PC for seed germination and root elongation toxicity test) in polystyrene Petri dishes (diameter 85 mm) covered with a nylon net (100 seeds/plate) (Leme and Marin-Morales, 2008). TiO_2_NP suspensions were replaced by fresh ones every 24 h to assure its bioavailability for the test system.

All experiments were carried out at 25 °C in the dark. The seed germination and root elongation toxicity tests were performed using triplicate plates per treatment (100 seeds/plate), while the other assays were carried out using a single plate per treatment (Lin and Xing, 2007; Leme and Marin-Morales, 2008).

#### Seed germination and root elongation toxicity test

The seed germination and root elongation toxicity tests were carried out according to the protocol described by Rank (2003). After 5 days of exposure, seed germination (number of seedlings) and root length were measured. Toxicity was expressed as the difference of seed germination and root elongation when compared to the NC. The results were statistically analyzed using a Shapiro-Wilk test followed by Student’s *t*-test (*p* < 0.05).

#### Cyto- and genotoxicity assessments

*Allium cepa* roots of 2 cm in length (~5 days) were fixed in a mixture of ethanol and acetic acid (3:1–v/v, Merck). The fixed roots were stained with Schiff’s reagent, as described by Feulgen and Rossenbeck (Mello and Vidal, 1978), and the slides were prepared using the meristematic region according to the protocol described by Leme and Marin-Morales (2008).

Cytotoxicity was assessed by recording the changes in the mitotic index (MI) of the meristematic cells. Genotoxicity was determined by scoring different types of chromosomal aberrations (CAs) and nuclear abnormalities (NAs). Micronucleated cells were also scored to determine the mutagenicity (Leme and Marin-Morales, 2008). Additionally, the mode of action of TiO_2_NP was defined based on the analysis of different types of CAs, which were grouped as clastogenic (chromosome bridges and breaks) or aneugenic (chromosomal losses, chromosomal delay) according to Leme *et al.* (2008).

These parameters were evaluated under a light microscope (Olympus BX-40- magnification - 400 x) and 10 slides per treatment were analyzed (500 cells/slide). The treatments were statistically compared using the non-parametric Kruskal-Wallis test followed by the Student-Newman-Keuls test (*p* < 0.05).

### Complementary tests

Complementary tests to the genotoxicity test were performed to confirm the toxic effects of TiO_2_NP and to visualize its behavior inside the cells.

In order to perform these tests, only the highest concentration (1000 mg/L) was chosen, because this concentration had already been shown to be genotoxicologically toxic, and the current objective was to confirm the toxicity and observe the effects caused to the plant system by the TiO_2_NPs.

#### Silver-stained nucleoli and nucleolar organizer region (Ag-NOR)

Ag-NOR staining and nucleoli analysis were carried out using fixed roots of 1000 mg/L TiO_2_NP treatment and an NC group, according to the previous protocol described by Mazzeo and Marin-Morales (2015). Thus, this analysis was used to verify whether the nucleolar pattern was altered after TiO_2_NP exposure.

Images were obtained using a motorized Axio Imager Z2 epifluorescence microscope (Carl Zeiss, Jena, Germany), equipped with an automated scanning V Slide (Metasystems, Altlussheim, Germany). Five thousand cells of both treatments were analyzed. This analysis was performed only on *A. cepa* interphase cells. The number of nucleoli per cell was determined by the counting tool implemented in Anati-Quanti software (Aguiar *et al.*, 2007), and their size was determined by Image J using a specific tool that measures the area of each nucleolus (Passoni *et al.*, 2014). The results were statistically analyzed using a Shapiro-Wilk test followed by a Student’s *t*-test (*p* < 0.05).

#### Morpho-anatomical analysis

Onion roots of 2 cm in length (~5 days) were fixed in Karnovsky solution (Karnovsky, 1965) for light microscopy (LM) and transmission electron microscopy (TEM) analyses. For both methodologies, visual analysis was performed.

#### Light microscopy (LM)

Fixed roots were dehydrated with ethanol, embedded in acrylic resin (methacrylate) and cut into 5 μm longitudinal sections (manual rotary microtome, Reichert). Sections were stained with toluidine blue stain at pH 4.0 (O’Brien and McCully, 1981) and the slides were sealed using Entellan^®^. Photographs of the stained sections were taken using a LM Zeiss Axioskop 2 equipped with a digital camera (MRC3). Five slides per treatment were analyzed.

#### Transmission electron microscopy (TEM)

Onion roots of 2 cm in length (~5 days) fixed in Karnovsky solution were rinsed in 0.05 M cacodylate buffer (3 x for 10 min) and post-fixed in 1% osmium tetroxide for 1 h. The samples were contrasted with uranyl acetate at 0.5%-v/v (overnight), dehydrated in acetone and embedded in resin (SPURR). After polymerization, ultrathin sections (70 nm) were placed on copper grids (300 mesh), counterstained with uranyl acetate and lead citrate (Reynolds, 1963), and observed under TEM (Jeol, JEM 1200EX-II) operating at 80 kV to avoid sample damage. The images were digitally captured by a CCD camera Gatan model Orius SC1000B.

#### Selected area electron diffraction (SAED)

Analyses of the TiO_2_NP crystal structure were made using the SAED technique with TEM (Jeol, JEM 1200EX II), and the electron diffraction figures were captured by a CCD Orius SC1000B camera. To determine the interplanar spacing of the crystal structure, the following expression was used: d (nm) = λL (nmpx)/D (px). In this formula, when measuring the distance (D) from the diffracted point to the center of the transmitted beam and knowing the camera constant (λL) and the TEM operating conditions, it is possible to measure the interplanar spacing (d), which defines certain characteristics of the crystalline material present in the sample. To determine the camera constant, which was 51.9 ± 0.2 nm pixel, a gold film was used. This technique was applied to *A. cepa* roots exposed to 1000 mg/L TiO_2_NP suspension to observe NP internalization. Additionally, this analysis was performed with the nanopowder form, obtained from 1000 mg/L TiO_2_NP suspension, before exposure to A. *cepa* roots.

## Results

### Physicochemical characterization of TiO_2_NPs

The nanopowder form was analyzed by transmission electron microscopy, as well as by X-ray diffraction. The analysis showed that the crystal structures of TiO_2_NPs comprise 100% anatase phase, consisting of 28.42% titanium and 71.58% oxygen. The DLS method revealed an average size of 45 nm and 107 nm for the aggregated particles, while the BET method showed a specific area of 83.47 m^2^/g.

**Figure 1:**
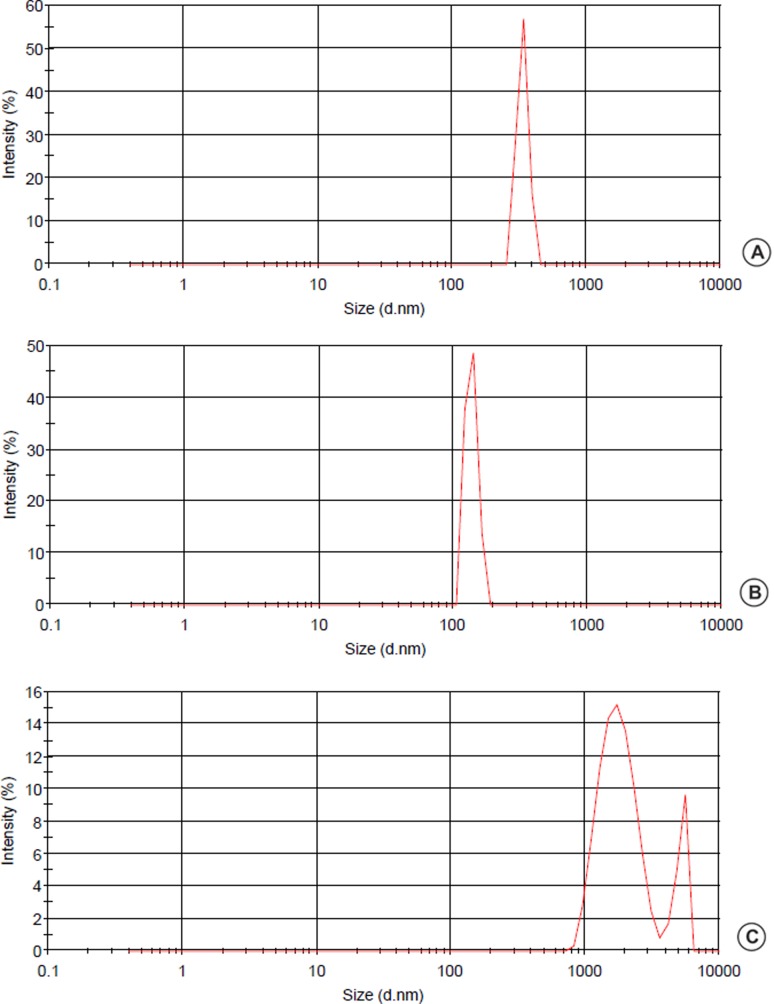
Intensity and size of TiO_2_NP suspensions tested, showing size distribution of the particles in nanometers. Graphics generated by Zeta Sizer Device (Malvern). (A) 10 mg/L TiO_2_NP, (B) 100 mg/L TiO_2_NP, (C) 1000 mg/L TiO_2_NP.

The analysis of the TiO_2_NP suspensions showed that their polydispersity indices were 91.1% (10 mg/L – pH 5.68), 76.5% (100 mg/L – pH 5.72), and 57.3% (1000 mg/L – pH 4.90). The size distribution of the TiO_2_NP suspensions is shown in Figure 1. The zeta potential values ranged from 21.2 to 2.99 mV, which confirms their instability.

The SAED pattern analysis of 1000 mg/L suspension showed that all TiO_2_NPs displayed a tetragonal format, which is a feature of the anatase phase, with an average size of 25 nm. On the other hand, the SAED analysis also revealed that the internalized TiO_2_NP in the vacuole compartment of meristematic cells shows an orthorhombic format, a feature of the brookite phase, with several sizes up to 450 nm.

### Seed germination and root elongation toxicity test

The results of the *A. cepa* toxicity test showed a significant reduction in both the germination rate (16–25%) and root development (11–18%) for all treatments with TiO_2_NP suspensions (concentration-dependent manner) (Table 1).

**Table 1:** Seed germination and root growth inhibition of *Allium cepa* test system exposed to titanium dioxide nanoparticles (TiO_2_NP).

Treatment	Concentration	SG	RL
	(mg/L)	M ± SD	M ± SD
NC	-	60 ± 1	1.93 ± 0.66
	10	53.3 ± 1.15*	1.60 ± 0.70*
TiO_2_NP	100	49.3 ± 0.57*	1.46 ± 0.77*
	1000	49 ± 0.57*	1.44 ± 0.64*
PC	6	7.66 ± 1*	0.580.17*

The tests were done with 100 seeds/plate, three replicates/treatment. NC: negative control, PC: positive control (ZnSO_4_.7H_2_O); SG: Seed Germination; RL: Root Length. M ± SD: mean ± standard deviation. Asterisk indicates significant difference to NC at *p* < 0.05 according to *t*-test.

### Cyto- and genotoxicity assessments

*A. cepa* meristematic cells exposed to the TiO_2_NPs suspensions showed a significant reduction in the MI at 100 mg/L and 1000 mg/L, and a tendency for the MI to decrease at the 10 mg/L concentration in a concentration-dependent manner. Significantly higher levels of both CA and MN were also observed after exposure of the *A. cepa* roots to TiO_2_NP at 1000 mg/L, showing a tendency to increase CA and MN at 10 mg/L and 100 mg/L (Table 2).

**Table 2:** Alterations in meristematic cells of *Allium cepa* exposed to different suspensions of titanium dioxide nanoparticles (TiO_2_NP).

Treatment	Concentration	MI	CA	MN
MN	(mg/L)	M ± SD	M ± SD	M ± SD
NC	-	246.9 ±1.55	0.70 ±0.67	0.60 ±0.69
TiO_2_NP	10	244.2 ±1.75	1.10 ±0.73	0.60 ±0.69
	100	240.7 ±1.15^*^	2.19 ±0.63	2.39 ±0.70
	1000	236.5 ±2.43^*^	5.28 ±1.06^*^	4.88 ±0.88^*^
PC	10	226.5 ± 1.90^*^	8.05 ±1.44^*^	33.3 ±2.42^*^

The tests were done with 5000 cells analyzed per treatment. MI: mitotic index, CA: chromosomal aberrations, MN: micronuclei, NC: negative control, PC: positive control – methyl methane sulfonate. M ± SD: mean ± standard deviation. Asterisks indicate significant differences at *p* < 0.05 compared to NC according to Kruskall-Wallis.

The analysis of the different types of CA is shown in Table 3. The main types of CAs were the chromosomal bridge, which was significantly higher than the NC at 100 and 1000 mg/L of TiO_2_NPs, and chromosomal breaks (significant frequencies for all tested concentrations). Delayed chromosomes, chromosomal adherence, and nuclear buds were also observed in the meristematic cells of *A. cepa* exposed to the TiO_2_NPs, and significantly higher frequencies were observed only at the highest tested concentration (1000 mg/L).

**Table 3:** Chromosome aberration (CA) types observed in meristematic cells of *A. cepa* exposed to titanium dioxide nanoparticles (TiO_2_NP).

CA	NC	TiO_2_NP (mg/L)			PC
		10	100	1000	
**Clastogenic**					
Chromosomal bridge	0.1	0.06	0.22*	0.36*	0.66*
Chromosomal breaks	0.02	0.08*	0.1*	0.36*	0.48*
Total	0.12	0.14	0.32	0.72	1.14
**Aneugenic**					
Chromosomal loss	0	0.02	0.02	0	0.02
Chromosomal delay	0	0	0.04	0.1*	0.04
Chromosomal adherence	0	0.02	0.06	0.16*	0.22*
Nuclear bud	0	0.04	0	0.08*	0.12*
Total	0	0.08	0.12	0.34	0.40

NC: negative control, PC: positive control – 10 mg/L methyl methane sulfonate. Data are expressed in frequency (%). Five thousand cells were analyzed per treatment. Asterisks indicate significant differences at *p* < 0.05 according to Kruskall-Wallis.

These results indicated the genotoxicity, mutagenicity, and cytotoxicity of TiO_2_NPs and were the starting point for complementary tests that confirmed the toxicity of these NPs and allowed to observe what happens inside the cells. All these tests were performed only at the highest concentration (1000 mg/L), since the objective was the observation of intracellular mechanisms. The results are reported below.

### Nucleolar organizer region (NOR) analysis

**Figure 2:**
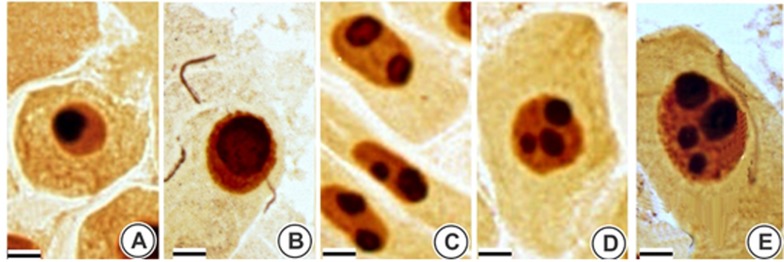
Meristematic cells of *Allium cepa* germinated in the negative control (NC) and 1000 mg/L TiO_2_NP subjected to Ag-NOR staining and banding. (A) Nucleoli germinated in NC, (B-E) cells germinated in 1000 mg/L TiO_2_NP, (B) increased size nucleoli, (C) two nucleoli, (D) three nucleoli, (D) four nucleoli. Scale bars 20 μm.

Significant increases in the NORs were observed in the *A. cepa* meristematic cells exposed to TiO_2_NP at 1000 mg/L (Figure 2). Moreover, the Ag-NOR staining data show a significant increase in the nucleolar score (1.50 ± 0.09 in NC to 1.82 ± 0.27 in TiO_2_NP) and average size of the nucleoli (2221 ± 159 in NC to 2516 ± 173 in TiO_2_NP) for the *A. cepa* cells exposed to TiO_2_NP compared with the NC (Student’s *t*-test, *p* < 0.05).

### Morpho-anatomical analysis

**Figure 3:**
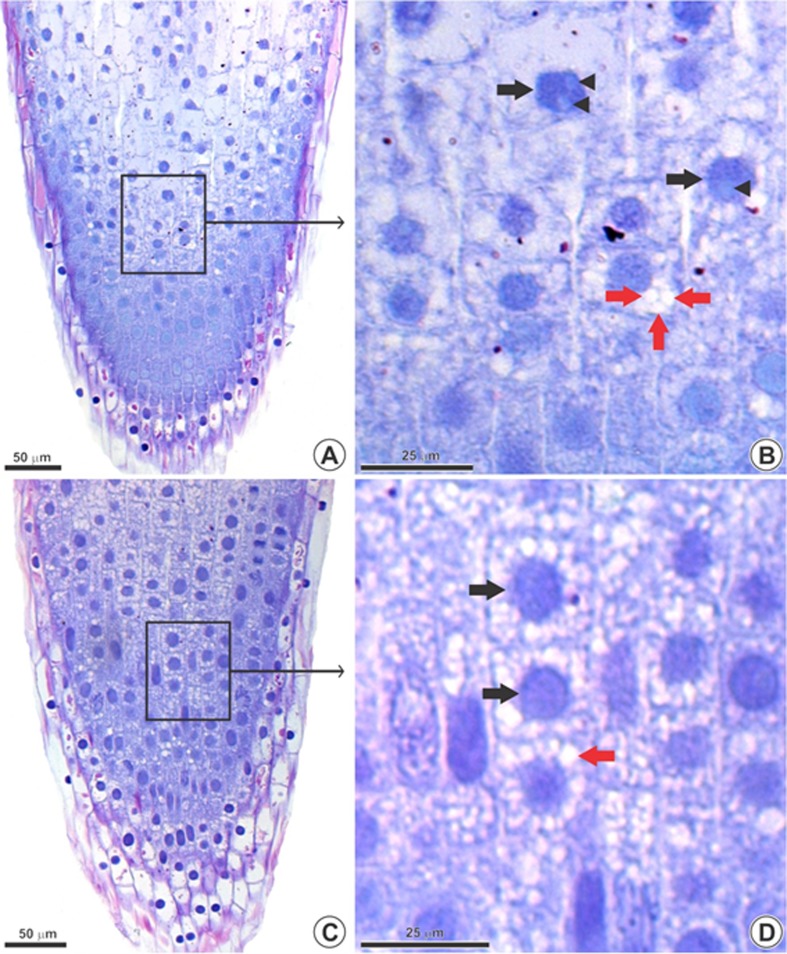
Root tip of *Allium cepa* (longitudinal sections) observed in light microscope. Negative control (A-B) and group exposed to 1000 mg/L TiO_2_NP (C-D). (A) Control root tip. (B) Selected region of the apical root meristem in A/B. (C) 1000 mg/L TiO_2_NP. (D) Selected region of the apical root meristem in C/D. Black arrow: nuclei; black arrowhead: nucleoli in evidence; red arrow: vacuoles increased in volume and number.

**Figure 4:**
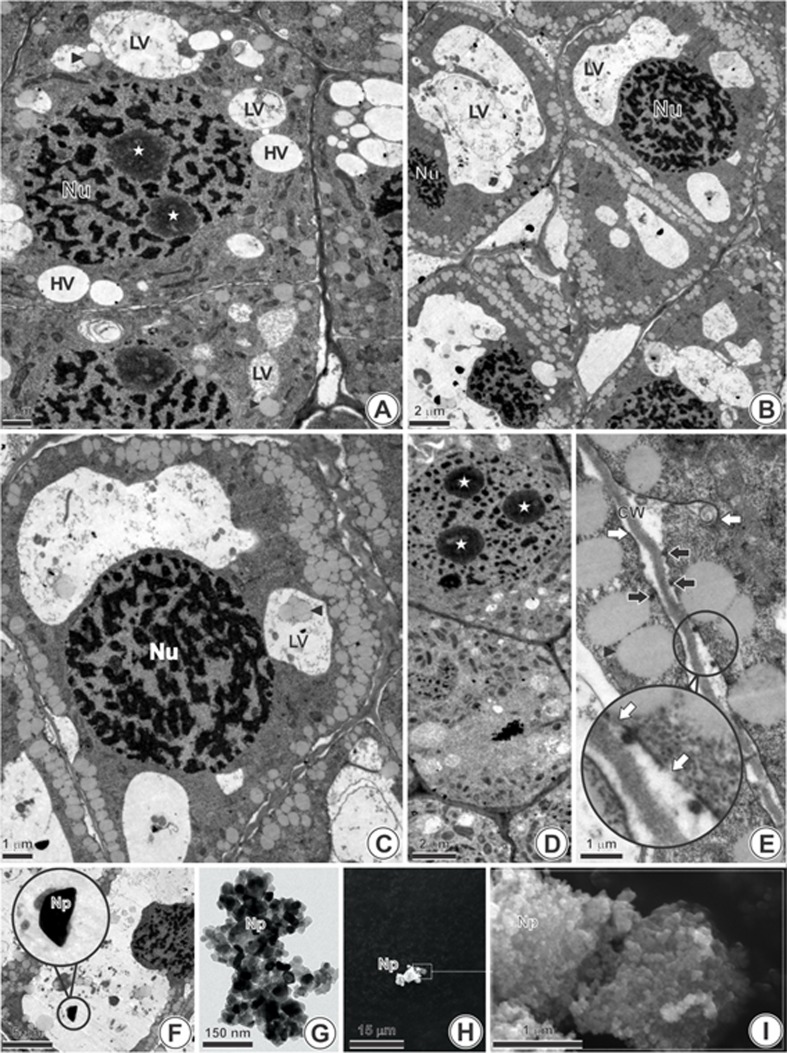
Root tip of *Allium cepa* (longitudinal sections) observed in transmission electron microscope. (A) Negative control and cells exposed to 1000 mg/L TiO_2_NP (B-E). Suspension of 1000 mg/L TiO_2_NP observed by selected area electron diffraction analysis (SAED) in transmission electron microscope (F-G) and scanning microscope (H-I). (A) Cell with lytic vacuole (LV) and hyaline (HV), and nucleus (Nu) with two nucleoli (star). (B) Increase in the volume and number of lytic vacuoles and increased volume of oil bodies (arrowhead). (C) Large number of oil bodies (arrowhead) in the adjacencies of plasma membrane and within the lytic vacuoles. (D) Nucleus with three nucleoli (star). (E) Rupture of the plasma membrane (white arrow) and electron-dense corpuscles associated with the plasma membrane (black arrow). Abbreviations: CW (cell wall). (F) Internalized TiO_2_NP in the vacuole with 450 nm in the brookite phase. (G) Characterization of the TiO_2_NP suspension in TEM. (H) Characterization of TiO_2_NP in scanning microscope – 500x. (I) Characterization of TiO_2_NP in scanning microscope, 50,000 x.

The LM analysis showed that the NC meristematic cells are thin-walled and relatively small. These cells contain numerous small vacuoles and large nuclei with one or two nucleoli (Figure 3A,B). With the TiO_2_NP treatment, the nuclei appear more condensed (Figure 3C,D) with up to three nucleoli (Figure 4D) and more and larger vacuoles.

The TEM analysis revealed that the meristematic cells from NC have organelles with peripheral disposition and a smooth cell wall. Additionally, two types of vacuoles can be characterized: with hyaline content, which are small, rounded, and have lytic vacuoles that have a higher amount of electron-dense material and lenticular shape. Oil bodies, isolated or associated with lytic vacuoles, were also observed in the cytoplasm of NC cells.

Rupture of the plasma membrane and a large number of oil bodies with peripheral disposition were observed in cells exposed to TiO_2_NP (1000 mg/L) (Figure 4B–E). In addition, *A. cepa* meristematic cells exposed to TiO_2_NP also exhibited a greater number of lytic vacuoles, which were larger (Figure 4E) compared to NC.

### Selected area electron diffraction (SAED)

**Figure 5:**
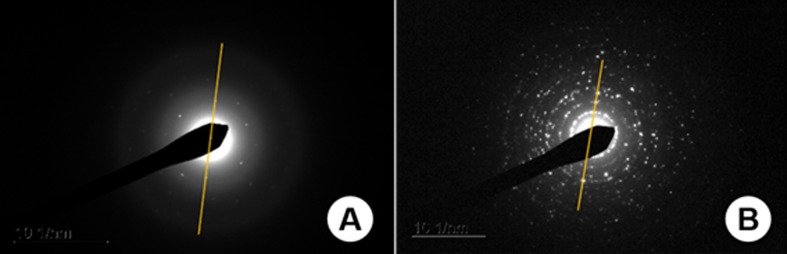
Selected area electron diffraction (SAED) pictures used in the calculation of interplanar distances. (A) TiO_2_ in brookite phase inside the roots of *A. cepa*. (B) TiO_2_ in anatase phase in nanopowder form used in this experiment.

The electron diffraction analysis performed in *A. cepa* roots exposed to 1000 mg/L of TiO_2_NPs revealed the presence of TiO_2_NPs inside lytic vacuoles in *A. cepa* cells. These particles had an approximate size of 450 nm and, according to the analysis of interplanar spacing and orthorhombic format, they were in the brookite phase. On the other hand, the SAED nanopowder analysis confirmed the manufacturer information saying that the TiO_2_NP in the powder form was 100% anatase phase and with an average size of 25 nm. Table 4 shows the interplanar distances (d) obtained through the analysis performed on the *A. cepa* roots and in the nanopowder form. Figure 4F–I shows the images of this analysis, and Figure 5 shows the two forms of TiO_2_NP (anatase and brookite phases).

**Table 4:** Analysis by selected area electron diffraction (SAED) in suspension and inside cells of *A*. *cepa* roots exposed to 1000 mg/L TiO_2_NP.

Material	Profile	D_measured_ (nm)	D_tabulated_ (nm)	Δ (%)
			Brookite phase	
	1	0.268 ± 0.04	0.2728	- 1.75
In *A. cepa* roots cells	1	0.132 ± 0.01	0.1335	- 1.12
	2	0.268 ± 0.04	0.2669	+ 0.41
	2	0.133 ± 0.01	0.1335	- 0.37
	3	0.228 ± 0.03	0.2295	- 0.65
	3	0.154 ± 0.02	0.1530	+ 0.65
			Anatase phase	
	1	0.344±0.004	0.352	-2.3
	2	0.237±0.002	0.2378	-0.3
Nanopowder	3	0.185±0.001	0.1892	-2.2
form	4	0.169±0.001	0.16999	-0.6
	5	0.0934± 0.0005	0.09464	-1.3
	6	0.0853± 0.0004	0.08464	+0.8

D_measured_ values are compared with D_tabulated_ values and from the difference (Δ) percentage, the phase identification is performed.

## Discussion

Most of the phytotoxicity studies of TiO_2_NPs do not comprise a characterization of their suspensions (*e.g.,* TiO_2_NPs suspensions), and only characterize the nanopowder (Ghosh *et al.*, 2010; Klancnik *et al.*, 2010; Kumari *et al.*, 2011; Pakrashi *et al.*, 2014). There is an imminent demand to elucidate the mechanisms of toxicity of NPs in plants in order to protect these key organisms of terrestrial and aquatic ecosystems, as they are the base of the food chain and support several ecosystems services, such as pollination. Within this context, the knowledge of NPs toxicity mechanisms can be better achieved when nanotoxicological studies include information about the features of NPs in suspension (Schwab *et al.*, 2016).

When in aqueous media, the pH of samples is considered one of the most important factors that may alter the values of the zeta potential (Jiang and Oberdorster, 2009). Zeta potential (ζ) is the measurement of the particle potential at the surface of the hydrodynamic shear. In this study, when analyzing the zeta potential values, all the suspensions presented instability in their colloidal systems, facilitating the formation of particle aggregates, even after sonication of these suspensions. Besides that, regarding the zeta potential, the existence of a pH value in which the values of the negative and positive charges are present in the same amount around the particles is known as point of zero charge (pH_pzc_). The pH_pzc_ of TiO_2_NPs in anatase phase is pH 6.3 (Finnegan *et al.*, 2007). In this case, TiO_2_NP is considered an acidic metal oxide, which means that its highly hydroxylated surface tends to donate protons by dissociating water, binding the OH^-^ ions and releasing H^+^ ions, leaving these NPs positively charged. Our TiO_2_NPs suspensions, at the highest concentration (1000 mg/L), whose pH was measured as 4.90, probably showed many particles with positive charges, which make the entrance of these NPs into cells possible by passing cell membranes. Additionally, the high polydispersity index of TiO_2_NPs in the three concentrations tested in this study, allowed the observation of particles of different sizes. This information is important because it indicates that particle size is not homogeneous, which may explain the damage caused by TiO_2_NPs, considering the existence of particles at the nanoscale.

Phytotoxicity is usually estimated by the seed germination and root elongation toxicity test. Our findings showed that TiO_2_NPs slightly inhibited seed germination and root growth; however, cellular and genetic damages were observed to meristematic cells of *A. cepa* after TiO_2_NP exposure. Studies have pointed out that determination of phytotoxicity by macroscopic parameters is not always accurate (Lin and Xing, 2007; Klancnik *et al*.*,* 2010; Castiglione *et al.*, 2016; Cox *et al.*, 2017), and genotoxicological analysis is required to predict the hazards of chemicals.

DNA damages to plant cells after NP exposure have been reported (Klancnik *et al.*, 2010; Kumari *et al.*, 2011; Pakrashi *et al.*, 2014; Castiglione *et al.*, 2016). In this study, increased frequencies of CA and MN in concentration-dependent manner were observed in *A.cepa* meristematic cells exposed to TiO_2_NPs, indicating their internalization. In addition, different types of CA were found. Significant values of clastogenic CAs (*e.g.,* chromosome breaks and bridges) were observed in all tested concentrations, while aneugenic CAs (*e.g.,* chromosome delay, chromosome adhesion, and nuclear buds) could be detected only at the highest concentration tested (1000 mg/L).

Chromosomal bridges result from structural changes between sister chromatids or between different chromosomes due to breaks or terminal deletions. Bridges that persist at the end of anaphase can originated chromosomal fragments (*i.e.*, breaks) during chromatin segregation (Humphrey and Brinkley, 1969). Chromosome breaks can also be caused by external agents, affecting the dynamics of the chromatin, and may damage the repair process (Terzoudi *et al.*, 2011).

Impairment of the mitotic spindle apparatus may lead to chromosomal adherences (Ventura-Camargo *et al.*, 2011). Adherence is an irreversible abnormality that involves the proteinaceous matrix of chromatin rather that DNA itself, usually leading to cell death (Fiskesjo, 1988). The interruption in mitotic spindle polymerization may promote a unilateral binding of the fuse to chromosomes, making their movement to the poles unfeasible and leading to chromosomal losses (Shamina *et al.*, 2003).

Chromosomal losses and breaks can originate in micronuclei and be from an aneugenic or clastogenic origin, while nuclear buds may originate from nuclear envelope formation prior to complete chromosome migration to the poles and their incorporation into the nuclei, as well as by cellular activities that promote the elimination of the amplified genetic material (Mazzeo *et al.*, 2011).

These genotoxicity results stimulated the accomplishment of complementary tests that allowed the observation of TiO_2_NPs internalization, which corroborated the toxicity results of this NP. These complementary tests were performed only with the highest concentration, because we aimed to understand TiO_2_NPs internalization and observe their effects. The significant increase in nucleolar score, as well as their sizes, in *A. cepa* interphase cells exposed to 1000 mg/L TiO_2_NP suggests that these results are due to an increase in genome activity (Mazzeo and Marin-Morales, 2015). According to Boulon *et al.* (2010), nucleoli are apparently major structures involved in the activation of cellular stress. Increased nucleoli volume suggests gene amplification and may be another indicative of genotoxicity (Mazzeo and Marin-Morales, 2015).

The cellular damage observed in *A. cepa* cells exposed to TiO_2_NPs indicates that this NP was taken up by the meristematic cells, causing deleterious effects. This study demonstrated that NPs were internalized by the meristematic cells of *A*. *cepa.* This internalization probably occurred due to the excess of positively charged particles that were easily attracted and internalized by the plasma membrane (negatively charged), allowing the observation of negative effects in these cells. These data agree with other studies of NPs (zinc oxide NPs and TiO_2_NPs) (Kumari *et al.*, 2011; Larue *et al.*, 2012), in which the authors also reported that NP uptake may result in damages from cellular defense mechanisms. The present results indicate that roots exposed to TiO_2_NPs show damage to both the plasma membrane and cell wall, suggesting that these barriers are not effective against TiO_2_NP uptake by meristematic cells.

Once the structure and function of both the cell wall and plasma membrane have been compromised, a physical barrier was formed by oil bodies located beneath them to reduce the uptake of TiO_2_NPs. According to Zhao *et al.* (2016), the increased number of oil bodies may indicate a cellular defense mechanism against toxicants. Moreover, a rise in the number and size of lytic vacuoles in the cytoplasm may also be related to this cellular defense mechanism, since this cellular compartment acts as a primary deposit site of toxic compounds (Ma *et al.*, 2015). Defense mechanisms are common when the plant encounters an adverse situation, like an exposure to a contaminant (Medina *et al.*, 2016; Car *et al.*, 2019). The SAED analysis showed the presence of TiO_2_NPs in lytic vacuoles of *A. cepa* meristematic cells that were exposed to this agent, as well as in *Triticum aestivum* spp (TiO_2_NPs at 100 mg/L), as observed by Larue *et al.* (2012).

The TEM analysis carried out on *A. cepa* cells exposed to 1000 mg/L TiO_2_NP showed that the deposit of this NP in lytic vacuoles occurs in aggregates of ca*.* 450 nm. The SAED results showed an orthorhombic structure compatible with the brookite phase, differently from a tetragonal structure expected for the anatase phase. This finding suggests that TiO_2_NPs were taken up by the cells and that a phase change had occurred (anatase to brookite).

According to the literature, the anatase and brookite phases are metastable and can switch their shape (Alotaibi *et al.*, 2018). The phase change showed in this work can be related to cellular defense mechanisms, as an attempt to minimize the damages caused by the internalized TiO_2_NPs. Therefore, the anatase to brookite phase conversion would be a way to “mitigate” the deleterious effects of TiO_2_NPs probably by reducing the damage mediated by reactive oxygen species (Larue *et al.*, 2012). It can be hypothesized that the interaction with biological molecules (*e.g.,* enzymes) produced by the plant system may be the responsible factor for the phase change (anatase to brookite) caused by TiO_2_NPs in *A. cepa* meristematic cells. However, further studies are needed to elucidate the crystalline phase change of TiO_2_NPs inside plant cells.

Finally, *A. cepa* was shown to be sensitive to the genotoxic and cytotoxic effects of TiO_2_NPs, thus being a suitable test system for predicting the hazard potential of NPs to plants. To minimize the toxic effects of TiO_2_NPs, plant cells exhibit cellular defense mechanisms that include increasing the number of oil bodies and lytic vacuoles. Furthermore, phase transformation of the crystal structure from anatase to brookite may also be an attempt to mitigate the toxic potential of TiO_2_NPs. In spite of the defense mechanisms, these NPs are still able to induce severe damages at nuclear (genotoxicity; changes in the nucleolar pattern) and cellular levels (cytotoxicity) in a concentration-dependent manner, which is indicative of their internalization. Probably, NPs were taken up by the meristematic cells through disruption of physical barriers (plasma membrane and cell wall). The phase conversion of TiO_2_NPs from anatase to brookite inside a plant cell was reported here for the first time, but the mechanisms associated with this change need to be elucidated by further studies.
